# Improved influenza viral vector based *Brucella abortus* vaccine induces robust B and T-cell responses and protection against *Brucella melitensis* infection in pregnant sheep and goats

**DOI:** 10.1371/journal.pone.0186484

**Published:** 2017-10-12

**Authors:** Aigerim Mailybayeva, Bolat Yespembetov, Sholpan Ryskeldinova, Nadezhda Zinina, Abylai Sansyzbay, Gourapura J. Renukaradhya, Nikolai Petrovsky, Kaissar Tabynov

**Affiliations:** 1 Laboratory of Infectious Disease Prevention, Research Institute for Biological Safety Problems, Zhambulskaya Oblast, Kordaiskiy Rayon, Gvardeiskiy, Republic of Kazakhstan; 2 Laboratory of Microbiology, Research Institute for Biological Safety Problems, Zhambulskaya Oblast, Kordaiskiy Rayon, Gvardeiskiy, Republic of Kazakhstan; 3 Food Animal Health Research Program, Ohio Agricultural Research and Development Center, Department of Veterinary Preventive Medicine, The Ohio State University (OSU), Wooster, United States of America; 4 Vaxine Pty Ltd and Flinders University, Bedford Park, Australia; Universidade Federal de Pelotas, BRAZIL

## Abstract

We previously developed a potent candidate vaccine against bovine brucellosis caused by *Brucella abortus* using the influenza viral vector expressing *Brucella* Omp16 and L7/L12 proteins (Flu-BA). Our success in the Flu-BA vaccine trial in cattle and results of a pilot study in non-pregnant small ruminants prompted us in the current study to test its efficacy against *B*. *melitensis* infection in pregnant sheep and goats. In this study, we improved the Flu-BA vaccine formulation and immunization method to achieve maximum efficacy and safety. The Flu-BA vaccine formulation had two additional proteins Omp19 and SOD, and administered thrice with 20% Montanide Gel01 adjuvant, simultaneously by both subcutaneous and conjunctival routes at 21 days intervals in pregnant sheep and goats. At 42 days post-vaccination (DPV) we detected antigen-specific IgG antibodies predominantly of IgG2a isotype but also IgG1, and also detected a strong lymphocyte recall response with IFN-γ production. Importantly, our candidate vaccine prevented abortion in 66.7% and 77.8% of pregnant sheep and goats, respectively. Furthermore, complete protection (absence of live *B*. *melitensis* 16M) was observed in 55.6% and 66.7% of challenged sheep and goats, and 72.7% and 90.0% of their fetuses (lambs/yeanlings), respectively. The severity of *B*. *melitensis* 16M infection in vaccinated sheep and goats and their fetuses (index of infection and rates of *Brucella* colonization in tissues) was significantly lower than in control groups. None of the protection parameters after vaccination with Flu-BA vaccine were statistically inferior to protection seen with the commercial *B*. *melitensis* Rev.1 vaccine (protection against abortion and vaccination efficacy, *alpha* = 0.18–0.34, infection index, P = 0.37–0.77, *Brucella* colonization, P = 0.16 to P > 0.99). In conclusion, our improved Flu-BA vaccine formulation and delivery method were found safe and effective in protecting pregnant sheep and goats against adverse consequences of *B*. *melitensis* infection.

## Introduction

Brucellosis is a chronic infectious disease of animals and humans. In infected pregnant animals the disease manifests as abortion. Due to social and economic impacts, brucellosis is included in the list of quarantine diseases. Ten species of *Brucella* are recognized as causative agents of brucellosis (*B*. *melitensis*, *B*. *abortus*, *B*. *suis*, *B*. *canis*, *B*. *ovis*, *B*. *neotomae*, *B*. *cetacea*, *B*. *pinnipedia*, *B*. *microti*, *B*. *inopinata*). Of these, brucellosis caused by *Brucella melitensis* in small ruminants (sheep and goats) is considered as the greatest risk to human health [1]. Approximately, 500, 000 people are infected by brucellosis each year. Despite the absence of a vaccine for humans, vaccination of animals against brucellosis is one of the most cost-effective measures for protecting the health of humans in endemic areas [2]; as well as an essential tool in eradication of the disease among farm animals [3]. Currently, brucellosis in sheep and goats is mainly prevented by using the live attenuated vaccine, *B*. *melitensis* Rev. 1 [4]. Although this vaccine is effective in controlling the disease, it has number of serious drawbacks as it can cause abortion in vaccinated pregnant animals, is virulent to humans and interferes with differential diagnosis of vaccinated animals from infected animals (DIVA) due to its induction of agglutinising antibodies. Furthermore, the strain Rev. 1 is resistant to the antibiotic streptomycin which is used to treat brucellosis. Though the problem of DIVA can be partially overcome by the conjunctival method of immunization and by avoiding vaccination of adult animals; these measures are difficult to follow and unrealistic in developing and underdeveloped countries [4, 5]. Therefore, development of a safe and effective vaccine against *B*. *melitensis* which could also has a DIVA potential is needed to solve the global problem of brucellosis.

Previously as a prophylactic measure against bovine brucellosis (*B*. *abortus*) we developed Flu-BA vaccine candidate in which a vector based on recombinant influenza virus subtypes H5N1 and H1N1 expresses the Brucella L7/L12 or Omp16 proteins from the NS1 (non-structural) gene open reading frame (ORF). In the past few years, we conducted many vaccine trials and demonstrated this vaccine’s safety and efficacy [6, 7]. Our results were comparable or even superior in vaccine trials comparing it to commercial vaccines made from *B*. *abortus* S19 and RB51 in cattle, including in pregnant heifers [8, 9]. We also demonstrated our candidate vaccine’s ability to induce prolonged protective immune response for up to 12 months [10], as well as showed its DIVA potential [7]. Furthermore, as the bovine Flu-BA vaccine is genetically stable, the vaccine virus does not excrete from the body into the environment, provides cross-protection against *B*. *melitensis* infection and is safe in contact humans [11, 12]. Hence our vaccine candidate meets the requirements of an "Ideal Brucellosis Vaccines" [13], as per the definitions of Schurig et al., [14] and Ko and Splitter [15]. These novel characteristics of our candidate Brucella vaccine made it an ideal vaccine for mass production and large scale application. Currently, our bovine Flu-BA vaccine is at the final stages of introduction into field application and commercialization in Kazakhstan.

The success in controling bovine brucellosis using influenza viral vectors expressing *Brucella* proteins together with, Montanide Gel01 adjuvant, served as a basis for analyzing this technology against brucellosis in sheep and goats caused by *B*. *melitensis*. In an earlier pilot study, the Flu-BA vaccine in sheep and goats provided 57.1% and 42.9% protection against *B*. *melitensis* infection, respectively [16]. However, this previous study was conducted in non-pregnant animals wherein brucellosis is not as highly pronounced compared to in pregnant animals, attributed to the presence of erythritol in the placenta, an important growth factor of *Brucella*. Therefore, it was expected that the efficacy of the Flu-BA vaccine in pregnant sheep and goats might be lower than in non-pregnant animals. Thus, our goal in this study was to maximize the effectiveness of the Flu-BA vaccine formulation for its application in pregnant sheep and goats. To achieve this task we incorporated several strategies in the vaccine formulation, including additional influenza viral vectors expressing Omp19 and Cu-Zn SOD proteins, increased the viral vector titer by ten-fold, increased the concentration of the adjuvant Montanide Gel01 by 2-fold, administered the vaccine simultaneously by subcutaneous and conjunctival routes and increased the number of doses to three. In this study, we report the immunogenicity and efficacy of our improved Flu-BA vaccine candidate in pregnant sheep and goats by comparison to the commercial *B*. *melitensis* Rev.1 vaccine.

## Materials and methods

### Generation of influenza viral vectors

All influenza viral vectors (IVV) were generated by a standard reverse genetics method using eight bidirectional plasmids pHW2000 as reported previously [11]. A total of eight influenza viral vectors in two viral subtypes expressing the *Brucella* L7/L12, Omp16, Omp19 or Cu-Zn SOD proteins from the open reading frame (ORF) of the *NS1* gene were generated: H5N1 (Flu-NS1-124-L7/L12-H5N1, Flu-NS1-124-Omp16-H5N1, Flu-NS1-124-Omp19-H5N1, Flu-NS1-124-SOD-H5N1) and H1N1 (Flu-NS1-124-L7/L12-H1N1, Flu-NS1-124-Omp16-H1N1, Flu-NS1-124-Omp19-H1N1 and Flu-NS1-124-SOD-H1N1). All influenza viral vectors of the subtypes H5N1 and H1N1 expressing *Brucella* proteins Omp19 and SOD were additionally included to the Flu-BA vaccine intended for use in cattle. The presence of appropriate Brucella protein insertions in the NS1 gene was confirmed by PCR and sequencing (data not shown). An improved vaccine formulation, including additional influenza viral vectors was provisionally referred as Flu-BA_Omp19-SOD.

### Vaccine preparation

Influenza vector vaccines (IVV) were prepared as described above in 10-day-old embryonated chicken eggs (CE; Lohmann Tierzucht GmbH, Cuxhaven, Germany) at 34°C for 48 h. The titer of the IVV was determined in CE as previously described [17]. The allantoic suspensions containing IVV H5N1 or H1N1 (titer of approximately 8.0 log10 EID_50_/ml) inserted with different Brucella antigenic genes were combined in a single pool at 1:1:1:1 ratio to obtain a tetravalent vaccine formulation. The mixtures of IVV (L7/L12, Omp16, Omp19 and SOD) were mixed in a 1:1 ratio with sterile stabilizing medium containing 12% peptone from casein (Sigma-Aldrich) and 6% saccharose (Sigma-Aldrich), mixed, aliquoted in 1 ml ampoules, lyophilized and stored at 2–8°C. Immediately before vaccination the lyophilized vaccine was resuspended (2.5 ml per ampoule) in a 20% solution of the adjuvant Montanide Gel01 (Seppic, Puteaux, France) in PBS.

### Vaccination and study design

**Fig 1:**
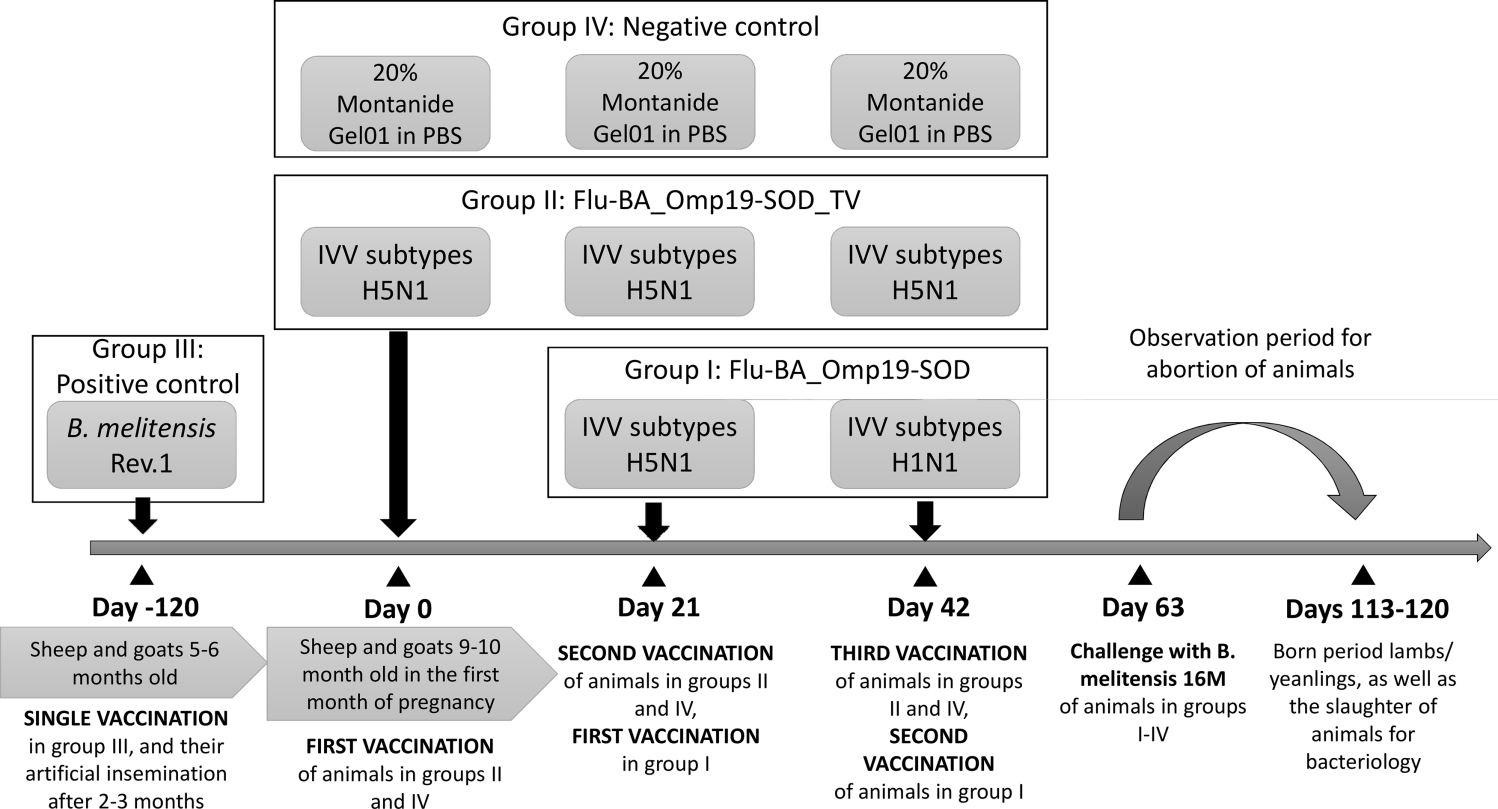
Experimental design.

A total of 40 Degeresskaya semifine meat and wool breed sheep and 39 Gorno-Altaisk breed goats aged 5–6 months procured from brucellosis free flocks were used in this study. All the animals were female and seronegative for brucellosis. A group of 5–6 months old sheep and goats (positive control Group III) received commercial *B*. *melitensis* Rev.1 vaccine (Antigen, Almaty, Kazakhstan; at dose 2.0 x 10^6^ CFU in 2.0 ml/animal) 3 months prior to artificial insemination once subcutaneously in the axillary region (right side) as per the manufacturer’s guidelines. This was carried out early because this vaccine causes abortions in some pregnant sheep and goats. The remaining sheep and goats at 8–9 months age were artificially inseminated at the synchronized estrus period and after one month they were examined for pregnancy by the hormonal method (progesterone). The pregnant sheep (n = 30) and goats (n = 30) were randomly assigned to four groups. Group (I) Flu-BA_Omp19-SOD (9 animals of each species); (II) Flu-BA_Omp19-SOD_TV (9 animals of each species); (III) Positive control (*B*. *melitensis* Rev.1 vaccine; 6 animals of each species); (IV) Negative control (Montanide Gel01 in PBS; 6 animals of each species). Group (I) animals were immunized twice concurrently via the subcutaneous (2.0 ml in the axillary region) and conjunctival (0.25 ml to each eye) routes at an interval of 21 days with vaccines generated from the IVV subtypes H5N1 (prime vaccination; 7.0 log_10_ EID_50_/animal) and H1N1 (booster vaccination; 7.0 log_10_ EID_50_/animal). Group II animals were vaccinated like group I, but using the vaccine generated from IVV subtypes H5N1 and administered three times at 21 days intervals. Sheep and goats in the negative control group were injected subcutaneously (2.0 ml in the axillary region) and conjunctivaly (0.25 ml to each eye) with 20% Montanide Gel01 adjuvant in PBS, three times at 21 day intervals (Fig 1).

The study was carried out in compliance with national and international laws and guidelines on animal handling. The protocol was approved by the Committee on the Ethics of Animal Experiments of the Research Institute for Biological Safety Problems of the Science Committee of the Ministry of Education and Science of the Republic of Kazakhstan (Permit Number: 0116/03). Animals were euthanized using sodium pentobarbital anesthetic and all recommended efforts were taken to minimize suffering.

### Assessment of vaccine safety

The safety of the Flu-BA vaccine in pregnant sheep and goats was determined in comparison with the positive (*B*. *abortus* Rev.1) and negative (Montanide Gel01 in PBS) control groups. The rectal temperature was recorded daily until 42 days post-vaccination (DPV) (group I), 63 DPV (groups II and IV) and 183 DPV (group III).

### Detection of hemagglutinin inhibiting (HAI) antibodies to influenza viral vectors

Serum antibodies to influenza virus vectors subtypes H5N1 and H1N1 in sheep and goats (groups I, II and IV) were determined on days 0, 21, 42 and 63 DPV by HAI assay as described previously using chicken red blood cell suspensions (1%) [17]. To remove non-specific inhibitors, blood samples were treated with the receptor-destroying enzyme from Vibrio cholerae (Denka Seiken Co. Ltd., Japan). The native influenza viral vectors subtypes H5N1 or H1N1 were used as the antigen at the working dose of four hemagglutination units.

### Assessment of vaccine immunogenicity

Serum samples (10 ml per Becton Dickinson Vacutainer tube) and whole blood (50 ml in tubes coated with EDTA/citrate) were collected from sheep and goats to determine antigen- specific humoral (IgG, IgG2a, IgG1 antibodies by ELISA; in groups I, II and IV) and T cell (stimulation index and IFN-γ production; in groups I, II and IV) responses in animals at 42 and 63 DPV.

### Antibody analysis

Ninety-six well microtiter plates (Nunc, Roskilde, Denmark) were coated overnight with pre-titrated mixture, as well as individual *Brucella* L7/L12, Omp16, Omp19 or SOD proteins (each at 2 μg/ml) in PBS, blocked for 1 h using PBS containing 1% ovalbumin (PBS-OVA; 200 μl/well), and washed with PBS containing 0.05% Tween-20 (PBS/Tw). Serial two-fold dilutions of the serum samples were diluted in PBS/OVA were added (100 μl/well) to the plates and incubated for 1 h at room temperature. A donkey anti-ruminant IgG horseradish peroxidase conjugate (Sigma, St. Louis, MO, USA) and monoclonal antibodies specific for sheep IgG1 and IgG2 (Novus Biologicals, Littleton, CO, USA) were used for detection. After a 90 min incubation at 37°C and washing, specific reactivity was determined by the addition of an enzyme substrate ABTS [2,2_azinobis(3-ethylbenzthiazolinesulfonic acid)] diammonium (Moss, Inc., Pasadena, CA, USA) at 100 ml/well. The absorbance values were measured at 415 nm. Antibody levels were expressed as the arithmetic mean ± S.D. of the OD obtained for sheep and goats included in each groups.

### Preparation of PBMC for lymphocyte proliferation assay

Peripheral blood mononuclear cells (PBMC) were isolated by density gradient centrifugation using a Ficoll-sodium diatrizoate gradient (DNA-Technology, Moscow, Russia) as previously described [16]. Cells number was adjusted to 10^7^ viable cells per ml determined by trypan blue dye exclusion, and 50 μl of each cell suspension (containing 5 x 10^5^ cells) was added to each of eight separate flat-bottomed wells of 96-well microtiter plates already plated with 100 μl of RPMI-1640 medium only or RPMI-1640 medium containing 8.0 μg of purified *Brucella* proteins L7/L12, Omp16, Omp19 or SOD per well. The cell cultures were incubated for 7 days at 37°C under 5% CO_2_. After incubation the cells were pulsed with 1.0 μCi of [^3^H] thymidine per well for 18 h. Cells were harvested onto glass filter mats and counted for radioactivity in a liquid scintillation counter. Cell proliferation results were converted to stimulation index (counts per minute [cpm] of wells containing antigens/cpm in the absence of antigens) for comparison.

### IFN-γ production

PBMC from each animal were adjusted to 10^7^ viable cells per ml as described previously. Aliquots (50 μl) of each cell suspension containing 5 x 10^5^ cells were added to the flat-bottomed wells of 96-well microtiter plates already plated with 100 μl of RPMI-1640 medium only or RPMI-1640 medium containing 8.0 μg of purified *Brucella* proteins L7/L12, Omp16, Omp19 or SOD per well. Cell cultures were incubated at 37°C under 5% CO2, and the supernatants were harvested 72 h later and assayed for IFN-γ using a commercial ELISA kit (RayBio^®^ Bovine IFN- γ ELISA Kit; RayBiotech, Inc., Norcross, GA, USA). This kit has been shown to cross-react with IFN-γ of sheep and goats [18]. Antigen-specific IFN-γ production was determined for each individual animal by subtracting the background concentration of IFN-γ in wells without antigen from the IFN-γ concentration in wells with antigen.

### Assessment of protective efficacy of the vaccine in sheep and goats

At DPV 42 (group I), 63 (group II and IV) and 183 (group III), pregnant sheep and goats at third month of pregnancy were challenged with a virulent strain of *B*. *melitensis* 16M at a dose of 10^6^ CFU/animal by subcutaneously (axillary region right side). Clinical observation of the challenged animals was performed for 50–57 days up to lambing or abortion. Animals that gave birth to non-viable lambs/yeanling were considered under the aborted group. From aborted fetuses or newborn lambs/yeanling within 12 h collected stomach content and spleen samples for bacteriological analysis. After abortion or lambing the adult animals were euthanized and aseptically collected samples of the lymph nodes (submandibular, retropharyngeal, right subscapular, left subscapular, mediastinal, bronchial, portal, para-aortic, pelvic, mesenteric and udder), parenchymal organs (liver, kidney, spleen and bone marrow) and placenta. In total, 16 organs were sampled from each animal. The tissue homogenates were plated onto *Brucella* agar plates and incubated at 37°C for 2 weeks, and the growth of bacterial colonies counted periodically during this time. The concentration of bacteria (CFU/g of tissue) in the tissue samples were determined by performing standard plate counts. An animal was considered to be infected if a *Brucella* colony was detected from the culture of one or more organs. The bacteriological examination was assessed by determining the effectiveness of vaccination (number of animals from which no *Brucella* colonies were isolated) and index of infection (number of animals from which Brucella was isolated from the organs and lymph nodes). All isolates were identified using routine methods as described previously [19].

### Statistical analysis

Difference in protections rates (protection from abortion and infection in pregnant sheep and goats and their fetuses or lambs/yeanling) between groups were compared by one-sided Fisher's exact test for two proportions at a significance level of *alpha* < 0.05. The differences in antibody levels (IgG, IgG1 and IgG2a), HAI titers, stimulation index, concentration of IFN-γ, index of infection and colonization of *Brucella* in tissues between groups was analyzed using two-way ANOVA followed by Tukey's or Sidak's multiple comparisons test. *P* values < 0.05 were considered significant. Means are reported with standard errors (SEM). The HAI assay data is given as Geometric mean titer (GMT) with a confidence interval of 95%. Statistical analyses of all experimental data were performed by using the Graphpad Prism Software, version 6.0 (Graphpad Software Inc., CA, USA).

## Results

### 1. Vaccine safety

Immunization of pregnant sheep and goats two- or three-times with the improved Flu-BA vaccine using simultaneous subcutaneous and conjunctival administration did not show any negative effect on the health status of the animals. The body temperature of the animals in all the experimental groups remained within normal limits (sheep 38.5–40.0°C, goat 38.5–40.5°C) during the entire period of the study (data no shown). Importantly, both the vaccines Flu-BA vaccine and *B*. *melitensis* Rev.1, and positive and negative control animals did not cause any abortion prior to bacterial challenge.

The only adverse event associated with the Flu-BA vaccine was a mild local reaction at the site of subcutaneous injection, induced by the Montanide adjuvant. All the animals in groups Flu-BA_Omp19-SOD and Flu-BA_Omp19-SOD_TV formed sterile infiltrates up to a diameter of 3 cm at one day post-vaccination, which completely resorbed by 2 months. These infiltrates did not affect the appetite, behavior or locomotor activity of animals. It should be noted that a similar local reaction was also observed in the negative control group (20% Montanide Gel01 in PBS), with infiltrates of up to a diameter of 2.5 cm that resolved completely by 1.5 months.

### 2. HAI antibody response to the influenza viral vectors

**Fig 2:**
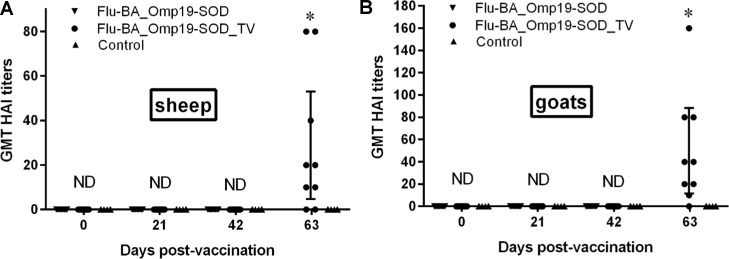
Geometric mean titers (GMT) of hemagglutination inhibition (HAI) antibodies against influenza virus subtypes H5 or H1 in serum samples of vaccinated pregnant sheep (A) and goats (B) at 0, 21, 42, 63 days post-vaccination (DPV). Pregnant sheep and goats in the group I (Flu-BA_Omp19-SOD) were immunized twice concurrently via the subcutaneous and conjunctival routes of administration at an interval of 21 days with vaccines generated from the influenza viral vectors (IVV) subtypes H5N1 (prime vaccination) and H1N1 (booster vaccination). The vaccination of animals of group II (Flu-BA_Omp19-SOD_TV) was carried out in the same way as in group I, but only the vaccine generated from IVV subtypes H5N1 was used, which was administered three times at 21 days intervals. Sheep and goats in the negative control group (IV) were vaccinated with 20% Montanide Gel01 adjuvant in PBS three times at 21 days intervals. Data are presented as GMT with 95% confidence interval; ND–not detected; * P = 0.0002 to P<0.0001 vs. appropriate control group; † P = 0.04 vs. Day 63 DPV of the Flu-BA_Omp19-SOD_TV sheep group. Statistical analysis was performed using two-way ANOVA followed by Sidak's multiple comparisons test. *P* values < 0.05 were considered significant.

HAI antibody titers against influenza virus subtypes H5 or H1 were undetectable (<1:10) in the serum samples of pregnant sheep and goats vaccinated with improved Flu-BA vaccine (groups I and II) and in the negative control group at day 21 after the first and second vaccination (Fig 2). But by DPV 63 HAI antibodies were detected to influenza viral subtype H5 only in Flu-BA_Omp19-SOD_TV vaccinated sheep and goats (P = 0.0002 to P <0.0001 vs. appropriate controls). HAI antibody titers were significantly higher in pregnant goats compared to sheep (50 [27–72.9] vs. 28.8 [5.9–51.8], 95% confidence interval, P = 0.04).

### 3. Antibody response to Brucella

**Fig 3:**
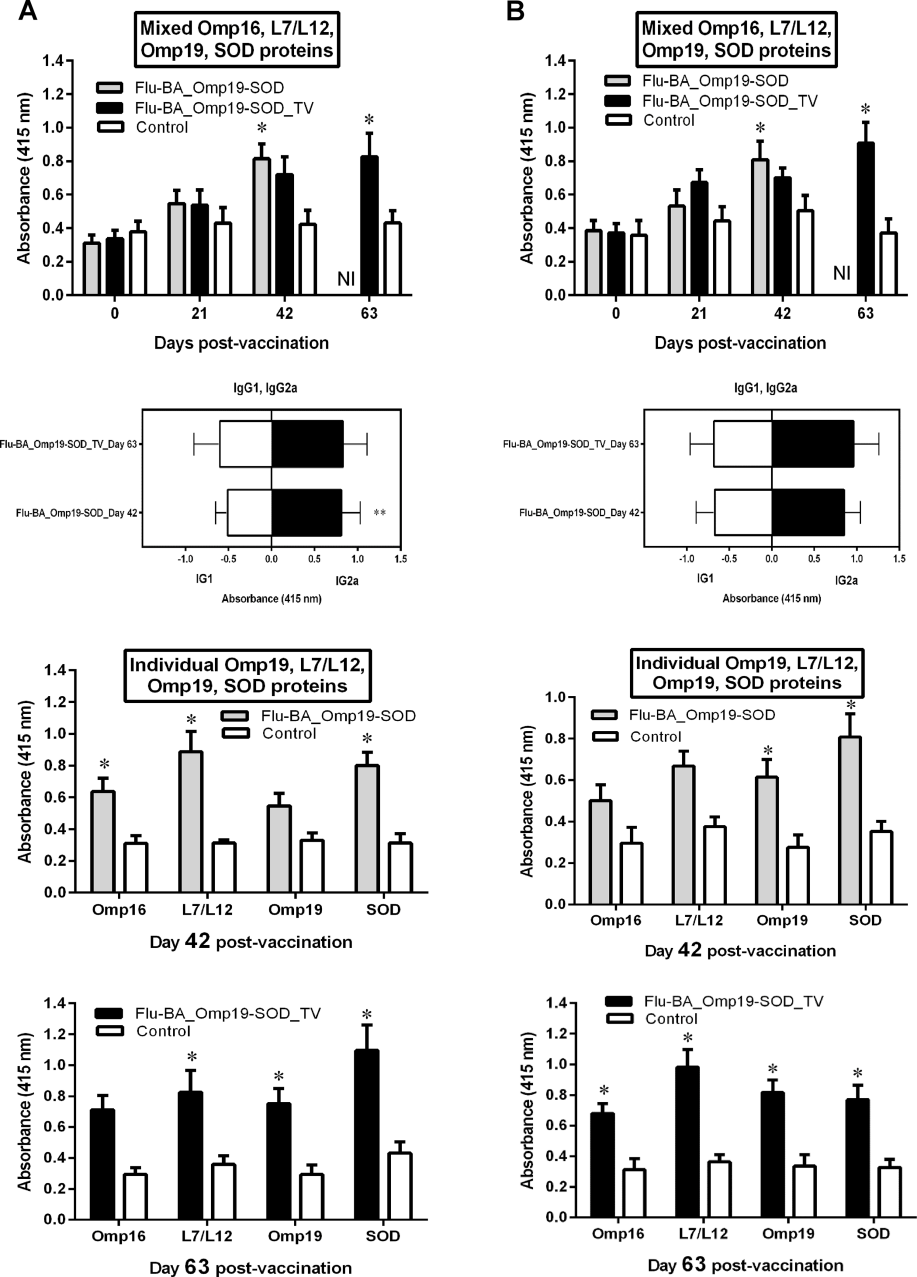
IgG, IgG1 and IgG2a antibody responses against total mixed and individual *Brucella* L7/L12, Omp16, Omp19 and SOD proteins in pregnant sheep (A) and goats (B) at 0, 21, 42, 63 days post-vaccination (DPV) by ELISA. Pregnant sheep and goats in the group I (Flu-BA_Omp19-SOD) were immunized twice concurrently via the subcutaneous and conjunctival routes at an interval of 21 days with vaccines generated from the influenza viral vectors (IVV) subtypes H5N1 (prime vaccination) and H1N1 (booster vaccination). The vaccination of animals of group II (Flu-BA_Omp19-SOD_TV) was carried out in the same way as in group I, but only the vaccine generated from IVV subtypes H5N1 was used, which was administered three times at 21 days intervals. Sheep and goats in the negative control group (IV) were vaccinated with 20% Montanide Gel01 adjuvant in PBS three times at 21 days intervals. Data are presented as optical density (OD) ± standard deviations; * *P = 0*.*04* to *P<0*.*0001* vs. appropriate controls; ** P = 0.04 vs. IgG1. Statistical analysis was performed using two-way ANOVA followed by Sidak's multiple comparisons test. *P* values < 0.05 were considered significant. NI—not investigated.

In the sera of the vaccinated sheep and goats (groups I and II) IgG antibody to a mixture of *Brucella* proteins L7/L12, Omp16, Omp19 and SOD began to appear from DPV 21, and it was significantly higher by DPV 42 (Flu-BA_Omp19-SOD vs. appropriate control, P = 0.04–0.008) and DPV 63 (Flu-BA_Omp19-SOD_TV vs. appropriate control, P = 0.007–0.0001) (Fig 3). Evaluation of the IgG antibody isotypes in the serum of sheep and goats revealed a slightly higher IgG2a levels over IgG1 with both immunization regimens. The specific IgG antibody response against individual *Brucella* proteins L7/L12, Omp16, Omp19 and SOD in serum samples collected at DPV 42 and 63 were significantly higher (P = 0.04 to P <0.0001 vs. appropriate controls).

### 4. Lymphocyte proliferation responses and IFN-γ production post-vaccination

**Fig 4:**
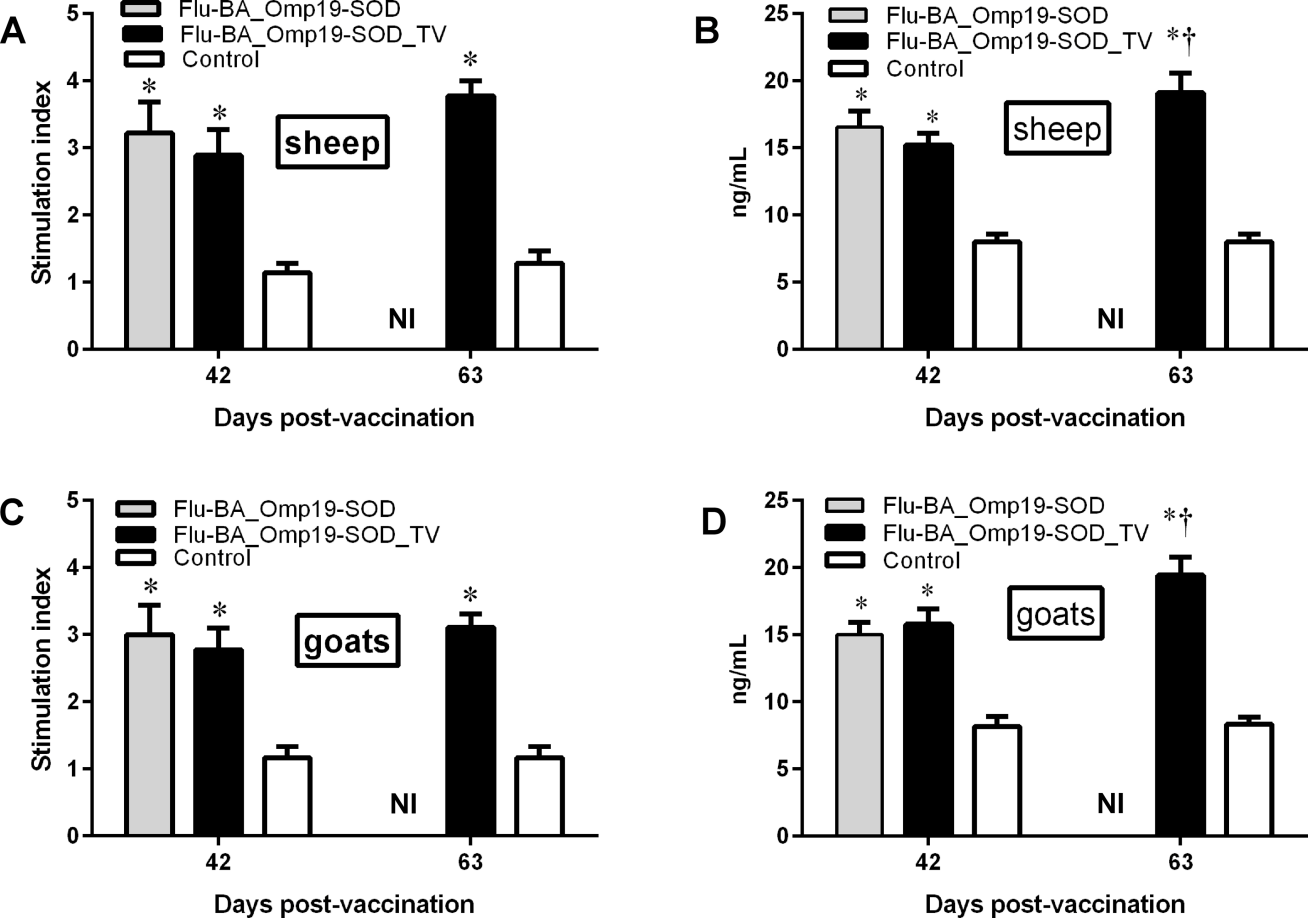
Lymphocyte stimulation index (A, C) and levels of IFN-γ (B, D) in the supernatants of PBMCs of pregnant sheep and goats at 42 and 63 days post-vaccination (DPV). Pregnant sheep and goats in the group I (Flu-BA_Omp19-SOD) were immunized twice concurrently via the subcutaneous and conjunctival routes at an interval of 21 days with vaccines generated from the influenza viral vectors (IVV) subtypes H5N1 (prime vaccination) and H1N1 (booster vaccination). The vaccination of animals of group II (Flu-BA_Omp19-SOD_TV) was carried out in the same way as in group I, but only the vaccine generated from IVV subtypes H5N1 was used, which was administered three times at 21 days intervals. Sheep and goats in the negative control group (IV) were vaccinated with 20% Montanide Gel01 adjuvant in PBS three times at 21 days intervals. All data are presented as mean ± standard error; * *P = 0*.*02 to P<0*.*0001* vs. appropriate control group; † *P = 0*.*02–0*.*01* vs. Day 42 DPV. Statistical analysis was performed using two-way ANOVA followed by Tukey's multiple comparisons test. *P* values < 0.05 were considered significant. NI—not investigated.

Analysis at DPV 42 of PBMC for antigen specific lymphocyte proliferation index and production of IFN-γ upon restimulation with the mixture of Brucella L7/L12, Omp16, Omp19 and SOD proteins, revealed a significantly higher lymphocyte stimulation index (*P = 0*.*02 to P<0*.*0001*) and IFN-γ secretion (*Р<0*.*0001*) in the pregnant sheep and goats (groups I and II) vaccinated with improved Flu-BA vaccine under either immunization regimes compared to the negative control group (Fig 4). The third dose of Flu-BA_Omp19-SOD_TV in pregnant sheep and goats resulted in a slight increase in the lymphocyte stimulation index, and a significant increase (P = 0.02–0.01 vs. Day 42 PIV) in IFN-γ production at DPV 63.

### 5. Protective efficacy against *B*. *melitensis* challenge infection

The protective efficacy against *B*. *melitensis* 16M infection of our candidate vaccine in pregnant sheep and goats was assessed using four parameters, namely protection against abortion, vaccination effectiveness or protection against infection (expressed in %), the infection index and colonization of *Brucella* in the organs and contents in the stomach of fetuses or spleen of lambs/yeanlings (Table 1). The results show that the improved Flu-BA vaccination under either immunization regimen provided protection against abortions in 66.7–77.8% of pregnant sheep and goats (*alpha* = 0.01–0.005 vs. appropriate controls). Abortions in all groups occurred in the third semester (121 to 133 days) of pregnancy. The level of protection against *B*. *melitensis* 16M infection (vaccination effectiveness) in vaccinated sheep (55.6–66.7%) and goats (44.4–55.6%), and their fetuses or lambs (77.8–90.0%)/yeanlings (70–72.7%) was also significant (*alpha* = 0.04–0.005, excluding goats in the Flu-BA_Omp19-SOD group, 44.4%), when compared with the control sheep and goats groups which had 100% abortion and 83.3–100% of their fetuses or lambs/yeanlings were infected.

**Table 1:** Rates of abortion, parturition and infection in the sheep and goats after challenge with the virulent strain *B*. *melitensis* 16M.

Group	Abortion, n ^a^ (%)	Parturition, n ^b^ (%)	Isolation of *B*. *melitensis*		Total	Isolation of *B*. *melitensis*		Total
			Positive (%)	Negative (%)		Positive (%)	Negative (%)	
***Sheep***						***Fetuses or lambs***		
Flu-BA_Omp19-SOD	3 (33.3)	6 (66.7)*	4 (44.4)	5 (55.6)*	9	2 (22.2)	7 (77.8)*	9
Flu-BA_Omp19-SOD_TV	2 (22.2)	7 (77.8)*	3 (33.3)	6 (66.7)*	9	1 (10.0)	9 (90.0)*	10 ^c^
*B*. *melitensis* Rev.1	0 (0)	6 (100)*	0 (0)	6 (100)*	6	0 (0)	7(100)*	7 ^c^
Control	6 (100)	0 (0)	6 (100)	0 (0)	6	5 (83.3)	1 (16.7)	6
***Goats***						***Fetuses or yeanling***		
Flu-BA_Omp19-SOD	3 (33.3)	6 (66.7)*	5 (55.6)	4 (44.4)	9	3 (30.0)	7 (70.0)*	10 ^c^
Flu-BA_Omp19-SOD_TV	3 (33.3)	6 (66.7)*	4 (44.4)	5 (55.6)*	9	3 (27.3)	8 (72.7)*	11 ^c^
*B*. *melitensis* Rev.1	0 (0)	6 (100)*	1 (16.7)	5 (83.3)*	6	0 (0)	7 (100)*	7 ^c^
Control	6 (100)	0 (0)	6 (100)	0 (0)	6	6 (100)	0 (0)	6

All isolates were identified as *B*. *melitensis* 16M.

^a^ Number of aborted sheep or goats, also includes animals that delivered a non-viable lambs or yeanling.

^b^ Number of sheep or goats that delivered a viable lambs or yeanling.

^c^ Some sheep and goats born twins.

* *alpha* = *0*.*04–0*.*001* vs. appropriate control group, one-sided Fisher's exact test.

**Fig 5:**
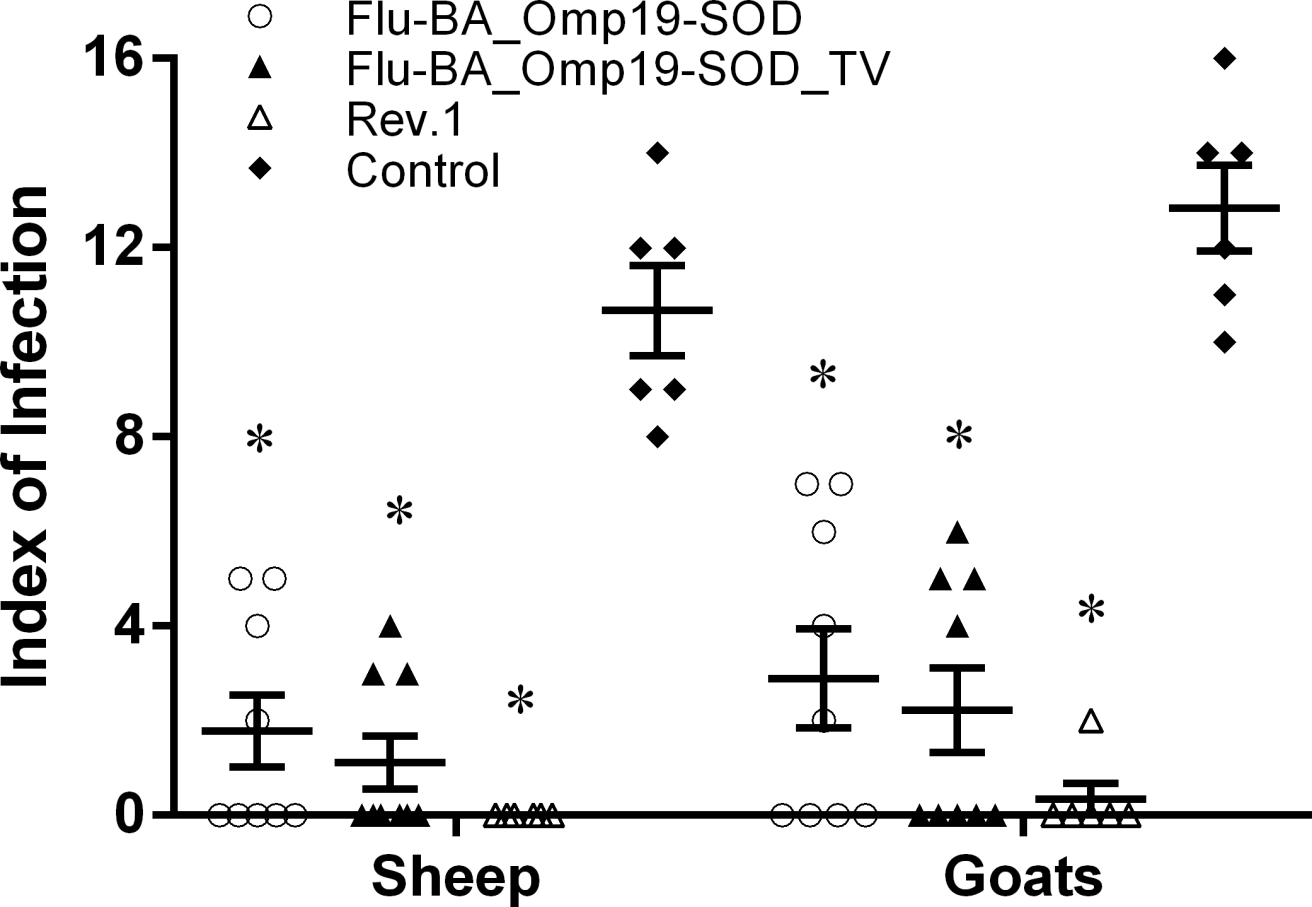
Index of infection for sheep and goats challenged with *B*. *melitensis* 16M at 113–120 days post-vaccination. Pregnant sheep and goats in the group I (Flu-BA_Omp19-SOD) were immunized twice concurrently via the subcutaneous and conjunctival routes of administration at an interval of 21 days with vaccines generated from the influenza viral vectors (IVV) subtypes H5N1 (prime vaccination) and H1N1 (booster vaccination). The vaccination of animals of group II (Flu-BA_Omp19-SOD_TV) was carried out in the same way as in group I, but only the vaccine generated from IVV subtypes H5N1 was used, which was administered three times at 21 days intervals. Sheep and goats in the negative control group (IV) were vaccinated with 20% Montanide Gel01 adjuvant in PBS three times at 21 days intervals. Animals in the positive control group (III) were immunized once subcutaneously in the axillary region (right side) with commercial vaccine *B*. *melitensis* Rev.1 according to the manufacturer's instructions. Challenge with the virulent strain *B*. *melitensis* 16M was performed via the subcutaneous route (10^6^ CFU/animal). The index of infection is the number of animals from which *Brucella* was isolated from the organs and lymph nodes. The data presented as mean ± standard error; * *P <0*.*0001* vs. appropriate control group IV. Statistical analysis was performed using two-way ANOVA followed by Tukey's multiple comparisons test. *P* values < 0.05 were considered significant.

The severity of *B*. *melitensis* 16 M infection in vaccinated sheep and goats and their fetuses or lambs/yeanlings of the Flu-BA_Omp19-SOD or Flu-BA_Omp19-SOD_TV groups as indicated by the index of infection (Fig 5; 1.1±0.5–2.8±1.0; *P <0*.*0001*) and rates of *Brucella* colonization in tissues (Table 2; 0.03±0.03–1.3±0.5 log10 CFU/g of tissue; *P = 0*.*04 - <0*.*0001*) were significantly lower than that of the control groups (index of infection 10.6±0.9 to 12.8±0.9; *Brucella* colonization 0.05±0.05 to 3.6±0.4 log10 CFU/g of tissue).

**Table 2:** Colonization and incidence of recovery of *B*. *melitensis* in tissues after challenge with *B*. *melitensis* 16M.

Sample type	Log10 mean ± SEM CFU/g of tissue (number recovered/total number)							
	*Sheep*				*Goats*			
	Flu-BA_Omp19-SOD	Flu-BA_Omp19-SOD_TV	*B*. *melitensis* Rev.1	Control	Flu-BA_Omp19-SOD	Flu-BA_Omp19-SOD_TV	*B*. *melitensis* Rev.1	Control
Submandibular LN	0±0 (0/9)	0±0 (0/9)	0±0 (0/6)	0±0 (0/6)	0±0 (0/9)	0±0 (0/9)	0±0 (0/6)	0.2±0.12 (2/6)
Retropharyngeal LN	0±0 (0/9)	0±0 (0/9)	0±0 (0/6)	0.46±0.21 (3/6)	0.15±0.1 (2/9)*	0.1±0.1 (1/9)*	0.05±0.05 (1/6)*	1.0±0.19 (6/6)
Right subscapular LN	0.49±0.21 (4/9)*	0.31±0.16 (3/9)*	0±0 (0/6)*	2.03±0.18 (6/6)	0.78±0.31 (5/9)*	0.64±0.28 (4/9)*	0.08±0.08 (1/6)*†	3.0±0.22 (6/6)
Left subscapular LN	0.06±0.06 (1/9)	0.03±0.03 (1/9)	0±0 (0/6)	0.58±0.39 (2/6)	0.06±0.06 (1/9)*	0±0 (0/9)*	0±0 (0/6)*	1.66±0.34 (6/6)
Mediastinal LN	0.17±0.11 (2/9)*	0±0 (0/9)*	0±0 (0/6)*	1.05±0.35 (4/6)	0.15±0.1 (2/9)*	0.1±0.1 (1/9)*	0±0 (0/6)*	1.23±0.39 (4/6)
Bronchial LN	0±0 (0/9)*	0±0 (0/9)*	0±0 (0/6)*	0.83±0.28 (4/6)	0.41±0.16 (4/9)*	0.37±0.2 (3/9)*	0±0 (0/6)*	1.28±0.41 (4/6)
Portal LN	0±0 (0/9)*	0±0 (0/9)**	0±0 (0/6)*	0.93±0.07 (6/6)	0.03±0.03 (1/9)*	0±0 (0/9)*	0±0 (0/6)*	1.48±0.18 (6/6)
Para-aortic LN	0±0 (0/9)*	0±0 (0/9)*	0±0 (0/6)*	0.85±0.2 (5/6)	0±0 (0/9)*	0.05±0.05 (1/9)*	0±0 (0/6)*	1.38±0.3 (5/6)
Pelvic LN	0±0 (0/9)*	0±0 (0/9)*	0±0 (0/6)*	0.95±0.31 (4/6)	0±0 (0/9)*	0.11±0.11 (1/9)*	0±0 (0/6)*	1.28±0.43 (4/6)
Mesenteric LN	0±0 (0/9)*	0±0 (0/9)*	0±0 (0/6)*	0.98±0.22 (5/6)	0±0 (0/9)*	0.05±0.05 (1/9)*	0±0 (0/6)*	1.51±0.31 (5/6)
Udder LN	0.52±0.27 (3/9)*	0.21±0.14 (2/9)*	0±0 (0/6)*	2.2±0.22 (6/6)	0.7±0.31 (4/9)*	0.23±0.15 (2/9)*	0±0 (0/6)*	2.78±0.24 (6/6)
Liver	0±0 (0/9)	0±0 (0/9)	0±0 (0/6)	0.16±0.16 (1/6)	0±0 (0/9)	0±0 (0/9)	0±0 (0/6)	0.45±0.17 (4/6)
Kidney	0±0 (0/9)	0±0 (0/9)	0±0 (0/6)	0±0 (0/6)	0±0 (0/9)	0±0 (0/9)	0±0 (0/6)	0.05±0.05 (1/6)
Spleen	0.4±0.24 (3/9)*	0.05±0.05 (1/9)*	0±0 (0/6)*	1.63±0.14 (6/6)	0.38±0.26 (2/9)*	0.13±0.1 (2/9)*	0±0 (0/6)*	2.76±0.14 (6/6)
Bone marrow	0±0 (0/9)	0±0 (0/9)	0±0 (0/6)	0±0 (0/6)	0±0 (0/9)	0±0 (0/9)	0±0 (0/6)	0±0 (0/6)
Placenta	1.08±0.52 (4/9)*	0.61±0.34 (3/9)*	0±0 (0/6)*†	3.61±0.43 (6/6)	1.31±0.53 (5/9)*	0.54±0.24 (4/9)*†	0±0 (0/6)*†	3.54±0.43 (6/6)
	***Fetuses or lambs***				***Fetuses or yeanling***			
Stomach content (fetuses) or spleen (lambs/ yeanling)	0.35±0.23 (2/9)*	0.14±0.14 (1/10)*	0±0 (0/7)*	2.0±0.43 (5/6)	0.61±0.31 (3/10)*	0.36±0.23 (3/11)*	0±0 (0/7)*	1.71±0.46 (6/6)

* P = 0.008 to P<0.0001 versus appropriate control group

† P = 0.04 to P<0.0001 versus appropriate Flu-BA_Omp19-SOD group, one-way ANOVA followed by Tukey’s multiple comparisons test.

The highest protection (100%) of pregnant sheep and goats from *B*. *melitensis* 16M infection was achieved in the group of animals vaccinated with *B*. *melitensis* Rev.1 (Tables 1 and 2, Fig 5). However, in all the tested parameters of protection, the *B*. *melitensis* Rev.1 vaccine had no statistically significant difference (protection against abortion and vaccination efficacy, *alpha* = 0.18–0.34, infection index, P = 0.37–0.77, *Brucella* colonization, P = 0.16 to P > 0.99) when compared to Flu-BA_Omp19-SOD_TV received animals. When compared to Flu-BA_Omp19-SOD vaccinated animals, *B*. *melitensis* Rev.1 vaccine had slightly lower level of colonization of *Brucella* in the right subscapular lymph nodes (in goats, P = 0.04) and placenta (in sheep and goats, P <0.0001).

## Discussion

Our earlier Flu-BA vaccine provided approximately 50% efficacy in vaccinated non-pregnant sheep and goats [16], which prompted us to evaluate the improved Flu-BA vaccine formulation and delivery system in pregnant sheep and goats against *B*. *melitensis* challenge infection. Differences in the improved Flu-BA vaccine was in the route of administration, dose of the vaccine, number of doses and concentration of the Montanide adjuvant. This was critical because, compared to bovine brucellosis (*B*. *abortus*) in cattle, *B*. *melitensis* infection in pregnant sheep and goats is highly virulent with severe clinical manifestations leading to abortions in most of the infected animals. Furthermore, in earlier trials the efficacy of the Flu-BA vaccine in pregnant cattle (70–80%) [9] was lower than in non-pregnant cattle (100%) [8]. Therefore, our goal in this study was to achieve maximum efficacy of Flu-BA vaccine in pregnant sheep and goats.

We used multiple approaches to improve the efficacy of Flu-BA vaccine in pregnant sheep and goats: (i) inclusion of additional influenza vaccine vectors (IVV) expressing Omp19 and Cu-Zn SOD proteins, which are immunodominant in *B*. *melitensis* like Omp16 and L7/L12 proteins in *B*. *abortus* [20–22]; (ii) ten-fold increase in the titer of IVV which helps in increasing the expression level of *Brucella* proteins; (iii) two-fold increase in the concentration of the adjuvant Montanide Gel01 from 10% to 20% which boosts the T-cell response [23, 8]; (iv) simultaneous subcutaneous and conjunctival administration of the vaccine was previously shown to improve the vaccine efficacy in cattle compared to the commercial *B*. *abortus* S19 vaccine [24]; (v) increased number of doses to three using IVV H5N1 (highly replicating virus) to boost the memory response of Flu-BA vaccine.

Initially, we compared the safety of our improved Flu-BA vaccine with two- and three immunization regimens in pregnant sheep and goats, with results consistent to that of our previous study [16], with absence of systemic reactions (overall general health condition, behavior, appetite and body temperature) and abortion. This Flu-BA vaccination outcome was critical as the commercial *B*. *melitensis* Rev.1 vaccination in pregnant sheep during the second and third trimester of pregnancy causes abortion in up to 80% of the animals [25]. Thus, in our study even the pregnant positive control sheep and goats were vaccinated with *B*. *melitensis* Rev.1 vaccine at three months before artificial insemination, to save the animals from any vaccine-induced abortions. Unlike in the earlier study [16], due to the increased concentration of the Montanide Gel01 adjuvant (10% vs. 20%) used in this study, all the improved Flu-BA and control adjuvant vaccinated animals had local reaction at the injection site.

The HAI antibody response plays an important role in protection against influenza [26]. Since our candidate Brucella vaccine has an IVV backbone, it is likely to induce an antibody response against the influenza virus in vaccinated sheep and goats, which may interfere with booster immunizations. We overcame this hurdle in our previous study in cattle by using a cross-immunization schedule [16], wherein IVV subtype H5N1 was used for prime vaccination and IVV subtype H1N1 for booster vaccination. Due to highly divergent HA protein in the virus subtypes the booster effect of Flu-BA vaccine was not compromised. In our initial study in sheep and goats we found that Flu-BA vaccine did not induce HAI antibodies to IVV subtypes H5N1 or H1N1 either after prime or booster vaccinations. Overall, due to attenuation of the IVV subtypes and weak immunogenicity due to poor cross-species specificity it failed to induce a strong immune response. To reconfirm this response, in this study we also included a group of pregnant sheep and goats vaccinated three times with IVV subtype H5N1 based Flu-Ba vaccine. Our results confirmed that HAI antibodies to IVV subtypes H5N1 or H1N1 were weak in the serum samples of sheep and goats vaccinated with both Flu-BA_Omp19-SOD and Flu-BA_Omp19-SOD-TV. This result was highly encouraging despite the fact that we used ten times more virus in the candidate vaccine (7.0 log_10_ EID_50_/animal vs. 6.0 log_10_ EID_50_/animal). Furthermore, in animals that received three doses of the vaccine Flu-BA_Omp19-SOD_TV, weak HAI antibody titers against H5N1 were detected only after the third dose of vaccination at DPV 63. Therefore, our results showed that the same IVV subtype backbone expressing Brucella proteins in sheep and goats could be used up to three immunizations at 21 days interval without affecting the vaccine efficacy. Our future research will be aimed at analyzing the repeatability of our results.

Brucella-specific humoral and especially cell-mediated responses are important for robust protection [27, 28]. Therefore, we evaluated both arms of the immune response in improved Flu-BA_Omp19-SOD_TV vaccinated pregnant sheep and goats, and found that at DPV 42 and 63 (after third dose) pronounced antigen-specific T cell response and IgG antibody responses to all the immunized *Brucella* proteins (L7/L12, Omp16, Omp19 and SOD). In addition, there was a slight predominance of specific IgG2a antibody isotype response over IgG1, suggesting a shift of the immune response towards the Th1 phenotype [15]. A significant IgG antibody response was observed against all individual *Brucella* proteins, indicating that all the four IVV including additional Omp19 and SOD were successfully expressed in the animals.

This study evaluated in pregnant sheep and goats protective efficacy against *B*. *melitensis* challenge infection of improved Flu-BA vaccine. We included the parameters characterizing both full protection (protection against abortion, vaccination effectiveness) of animals from *B*. *melitensis* 16M infection, and severity of the infection (infection index, colonization of Brucella from lymph nodes and organs) in animals. For parallel comparison purpose, we also included the commercial *B*. *melitensis* Rev.1 vaccine received animals. Our overall results, to the Flu-BA_Omp19-SOD_TV vaccine showed protection against abortion in 66.7 and 77.8% of animals and protection against *B*. *melitensis* 16M infections in 55.6 and 66.7% (and 72.7–90.0% of their fetuses or lambs/yeanlings) of sheep and goats, respectively. Even in diseased animals the infection index (lower than 4.5–9.6 times) and the level of colonization of Brucella in tissues of sheep and goats were significantly lower (more than 200 times) in comparison to negative control groups.

Importantly, results of the improved Flu-BA triple immunization regime in pregnant sheep and goats were not statistically different to the commercial *B*. *melitensis* Rev.1 vaccine. Thus, we can conclude that the best immunization regime for the improved Flu-BA vaccine is a triple administration of the vaccine at 3 weeks interval. Also the Flu-BA_Omp19-SOD vaccine generated from IVV subtype H5N1 backbone has many practical production conveniences such as: (i) significantly reduced production time, materials and labor resources, making the product cheaper and competitive; (ii) it makes the vaccination process convenient for field veterinarians, as this will not cause any likely confusion while using both H1N1 and H5N1 backbone Flu-BA vaccines formulations. It should also be noted that the protective efficacy in sheep and goats is not inferior [29, 30], and in fact even superior [31] to other vaccine candidates made of mutant strains of *B*. *melitensis* in R or S form. In earlier trials using commercial brucellosis vaccines in different types of animals, a positive effect with *B*. *melitensis* Rev.1 vaccine was obtained in cattle against *B*. *melitensis* infection [32], and in some trials negative results when *B*. *abortus* RB.51 vaccine was used in sheep and goats against *B*. *melitensis* infection [33].

In conclusion, our improved Flu-BA vaccine candidate formulation administered three times by simultaneous subcutaneous and conjunctival routes in pregnant sheep and goats elicited robust antigen-specific humoral and cell-mediated immune responses, resulting in protection in approximately 70% of pregnant animals against *B*. *melitensis* 16M infection. Future studies using the improved Flu-BA vaccine will be aimed at determining the duration of protective immunity in pregnant sheep and goats following the triple immunization protocol.
